# Structure and transport properties of triple-conducting Ba_*x*_Sr_1−*x*_Ti_1−*y*_Fe_*y*_O_3−*δ*_ oxides

**DOI:** 10.1039/d0ra10048j

**Published:** 2021-06-01

**Authors:** T. Miruszewski, K. Dzierzgowski, P. Winiarz, D. Jaworski, K. Wiciak-Pawłowska, W. Skubida, S. Wachowski, A. Mielewczyk-Gryń, M. Gazda

**Affiliations:** Gdańsk University of Technology Narutowicza 11/12 80-233 Gdańsk Poland tadeusz.miruszewski1@pg.edu.pl +48 58 348 66 12

## Abstract

In this work, Ba_*x*_Sr_1−*x*_Ti_1−*y*_Fe_*y*_O_3−*δ*_ perovskite-based mixed conducting ceramics (for *x* = 0, 0.2, 0.5 and *y* = 0.1, 0.8) were synthesized and studied. The structural analysis based on the X-ray diffraction results showed significant changes in the unit cell volume and Fe(Ti)–O distance as a function of Ba content. The morphology of the synthesized samples studied by means of scanning electron microscopy has shown different microstructures for different contents of barium and iron. Electrochemical impedance spectroscopy studies of transport properties in a wide temperature range in the dry- and wet air confirmed the influence of barium cations on charge transport in the studied samples. The total conductivity values were in the range of 10^−3^ to 10^0^ S cm^−1^ at 600 °C. Depending on the barium and iron content, the observed change of conductivity either increases or decreases in humidified air. Thermogravimetric measurements have shown the existence of proton defects in some of the analysed materials. The highest observed molar proton concentration, equal to 5.0 × 10^−2^ mol mol^−1^ at 300 °C, was obtained for Ba_0.2_Sr_0.8_Ti_0.9_Fe_0.1_O_2.95_. The relations between the structure, morphology and electrical conductivity were discussed.

## Abstract 2

Ba_*x*_Sr_1−*x*_Ti_1−*y*_Fe_*y*_O_3−*δ*_-based perovskite materials with different barium and iron contents are reported as triple conducting oxides (TCOs), which may conduct three charge carriers: oxygen ions, protons and electrons/holes.

## Graphical abstract

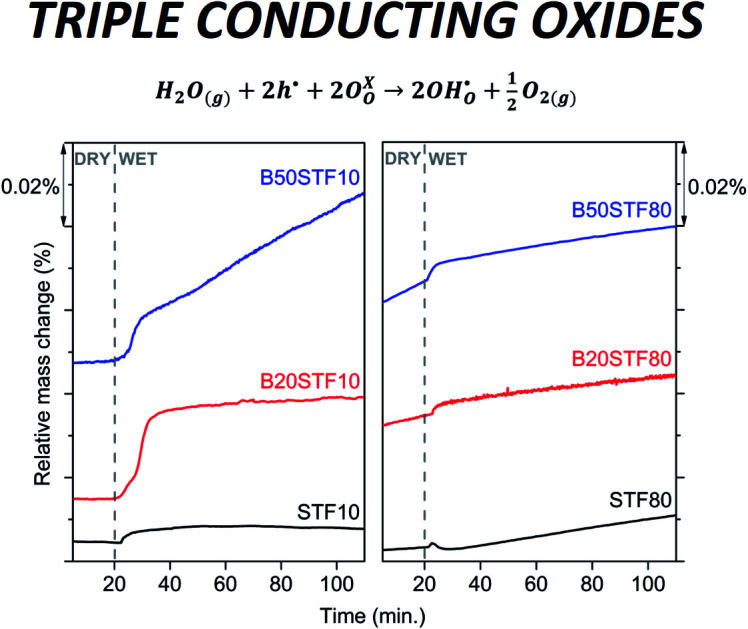

## 1. Introduction

Recently, triple conducting oxides (TCOs) with relatively high electronic, ionic and protonic conductivities have attracted a lot of attention.^1–3^ Such materials are promising electrode materials in protonic ceramic fuel cells (PCFCs) and electrolyzers (PCEs).^4^ The most promising candidates for single-phase TCOs are materials from the groups of doped perovskites like BaCe_0.4_Sm_0.2_Fe_0.4_O_3−*δ*_, layered perovskites PrBa_0.5_Sr_0.5_Co_2_O_5+*δ*_ or NdBa_0.5_Sr_0.5_Co_1.5_Fe_0.5_O_5+*δ*_ (NBSCF)^3,5,6^ and oxides belonging to the strontium titanate SrTiO_3_ (STO)–strontium ferrite SrFeO_3_ (SFO) solid solution system.

Strontium titanate is known semiconductor with a wide bandgap (*E*_g_ = 3.2 eV at 0 K) with low catalytic activity for oxygen reduction reaction and good chemical stability in a wide oxygen partial pressure range.^7^ Change of oxygen partial pressure (*p*_O_2__) from 10^−25^ to 10^−5^ atm. at 800 °C can transform SrTiO_3_ from an n-type into a p-type electronic conductor.^8^ To increase electronic and/or ionic conductivity, strontium titanate may be substituted at the strontium and titanium sites. Charge compensation by electrons and cation vacancies may be accomplished by introducing a donor dopant into Sr- (*e.g.* Y, La, Pr)^9–11^ and/or Ti- (*e.g.* Nb)^12,13^ sublattices, while the addition of acceptor dopants (Fe, Cr, Sc, Al, Co, Zn)^14–21^ leads to the formation of oxygen vacancies or electron holes.

Strontium ferrite is a mixed ionic–electronic conductor (MIEC) with relatively high metal-like conductivity (∼10^3^ S cm^−1^ at 850 °C) and high oxygen ion conductivity (∼0.2 S cm^−1^ at 850 °C) in air.^22^ The structure of SrFeO_3−*δ*_ strongly depends on the oxygen content, where the change of oxygen partial pressure may lead to a phase change from the orthorhombic brownmillerite structure to tetragonal or cubic perovskite structures.^23^

Solid solution of SrTiO_3_ and SrFeO_3_ namely, SrTi_1−*x*_Fe_*x*_O_3−*δ*_ (STF_*x*_), is a mixed ionic–electronic conductor. Substitution of titanium tetravalent cations in strontium titanate by iron cations leads to the formation of oxygen vacancies in the oxygen sublattice and influences the electrical properties of the material. Under the atmosphere with high *p*_O_2__ (oxidizing atmosphere), trivalent Fe^3+^ and tetravalent Fe^4+^ are predominant. On the other hand, in reducing atmospheres, Fe^3+^ and Fe^2+^ are the cations that are predominant. The electronic conduction is caused by the exchange of electrons or holes between the ions of different valence.^8,15^ In our previous report, the water uptake measurements showed a relatively low proton concentration in SrTi_1−*x*_Fe_*x*_O_3−*δ*_ for *x* < 0.5. The highest proton concentration was found in SrTi_0.9_Fe_0.1_O_2.8−*δ*_ at 400 °C. In this sample, the proton concentration was estimated as 5.2 × 10^−2^ mol mol^−1^, whereas in the samples with higher iron content the proton concentration was lower.^24^ To improve the water uptake for ABO_3_ materials, additional dopants at A- and B-sites should be introduced. Such substitutions were previously introduced for SrFeO_2.5_, where both the A-site and B-site various dopants were investigated in terms of hydration of the samples. For example, Raimondi *et al.* investigated the substitution on the Fe-site by cations with different sizes such as Y^3+^ or Zn^2+^ or the substitution of Ba^2+^ by the smaller Sr^2+^ cation. They concluded that such a substitution may strongly promote water uptake.^25^ A relatively high proton concentration was observed in barium and zinc-containing perovskite materials. For instance, the highest reported proton concentration for triple conducting perovskites was around 0.1 mol mol^−1^ for Ba_0.95_La_0.05_Fe_0.8_Zn_0.2_O_2.4_ at 250 °C.^21^ Introducing barium into the strontium sublattice of Ba_*x*_Sr_1−*x*_Ti_1−*y*_Fe_*y*_O_3_ (BSTF) ceramics lowered the bond strength and decreased the enthalpy of reduction and bandgap.^26,27^ Therefore, it seems that the introduction of barium into SrFeO_3_–SrTiO_3_ solid solution could be beneficial for triple conductivity for the following reasons: (1) increased concentration of oxygen vacancies due to smaller enthalpy of reduction should be beneficial both for oxygen ionic and protonic conductivity, (2) electronic conductivity may increase owing to reduced bandgap and (3) Ba doping should increase water uptake as indicated by the literature data.^25,28–30^

In this work, the influence of Ba content on the properties of Ba_*x*_Sr_1−*x*_Ti_1−*y*_Fe_*y*_O_3−*δ*_ oxides has been examined. The main effort was devoted to the studies of the structure, water uptake and electrical properties in two groups of materials: Ba_*x*_Sr_1−*x*_Ti_0.9_Fe_0.1_O_3−*δ*_ and Ba_*x*_Sr_1−*x*_Ti_0.2_Fe_0.8_O_3−*δ*_, which represent barium-substituted strontium titanate and ferrite structures.

## 2. Experimental

### 2.1. Synthesis of materials

The Ba_*x*_Sr_1−*x*_Ti_1−*y*_Fe_*y*_O_3−*δ*_ samples with *x* = 0, 0.2, 0.5 and *y* = 0.1, 0.8, were synthesized by a two-step solid-state route. Stoichiometric amounts of BaCO_3_, SrCO_3_, TiO_2_ and Fe_2_O_3_ powders (Sigma Aldrich, >99.9% purity) were mixed in an agate mortar for 30 min. Mixed powders were uniaxially pressed into 12 mm diameter round pellets under 400 MPa pressure. The pellets were calcined at 1000 °C in air for 12 h (3 °C min^−1^ heating/cooling rate). In the next step, the pellets were crushed, ground, re-pelletized under 400 MPa pressure and sintered at 1100 °C for 24 h. In the further text, the samples will be denoted as B*X*STF*Y*, where *X* and *Y* are the molar percentages of barium and iron, *e.g.* Ba_0.5_Sr_0.5_Ti_0.9_Fe_0.1_O_3−*δ*_ – B50STF10.

### 2.2. Structural and microstructural characterization

Crystal structure measurements at room temperature were performed by X-ray diffraction (XRD) method using Phillips X'Pert Pro diffractometer with Cu K_α_ (1.542 Å). The measurements were carried out in the angular range of 10–125°. XRD data were analysed with FullProf Suite software, where the unit cell parameters were calculated utilizing Rietveld refinement.^31^ As an initial point of the analysis, the unit cell parameters of the Ba_0.5_Sr_0.5_Ti_0.876_Fe_0.081_O_3−*δ*_ structure (*P*4/*mmm*, space group no. 123)^32^ and the SrTi_0.2_Fe_0.8_O_3−*δ*_ unit cell (*Pm*3̄*m* space group no. 221)^33^ were used.

The microstructure of the cross-sections of the sintered pellets was examined using FEI Quanta FEG 250 Scanning Electron Microscope (SEM). SEM images were collected using simultaneously with two detectors: the Everhart–Thornley Detector (ETD) for secondary electrons (SE) and the Backscattered Electrons Detector (BSED). The imaging was performed in high vacuum with 20 kV acceleration voltage. The density and porosity of the pellets were determined using the Archimedes method with kerosene as a liquid medium.

### 2.3. Electrochemical impedance spectroscopy (EIS)

For electrical measurements, platinum electrodes were ink painted (ESL 5542) and heated at 930 °C for 3 h. Typical dimensions of pellets used in electrical measurements were 4 × 2 × 2 mm^3^. Electrochemical Impedance Spectroscopy (EIS) measurements were performed in the frequency range from 1 Hz to 1 MHz. The signal amplitude varied from 10 mV (for the STF80 and B50STF80 samples) to 1 V (for the STF10 sample). The measurements were carried out in both wet synthetic air (*p*_O_2__ ≈ 0.195 atm., *p*_H_2_O_ ≈ 2 × 10^−2^ atm.) and dry composition of synthetic air and argon (*p*_O_2__ ≈ 0.195 atm., *p*_H_2_O_ ≈ 3 × 10^−5^ atm.) to compensate the oxygen partial pressure difference between dry and wet gas mixture. Impedance spectra were collected with Gamry Reference 600+ in the temperature range from 400 °C to 800 °C with 50 °C steps. At each temperature step, the samples were maintained until the thermodynamic equilibrium was achieved. The impedance spectra were analysed with ZView software.^34^ The obtained results were fitted with an equivalent circuit, which consisted of three RQ elements. The first element was fitted to include the influence of “negative resistance” phenomena. The “negative resistance” phenomena were observed for high frequencies in all 4-wire measured spectra's in BXSTF10. This artefact may appear in the case of four-electrode electrochemical impedance spectroscopy (EIS) measurements and it has been taken into consideration during the calculations of the sample resistances.^35^ The second RQ (large semicircle in Fig. 4) corresponds to the conductivity of the sample. The third RQ visible as a small semicircle is related to the processes occurring at the sample–electrode interface due to its high capacitance – 1 × 10^−6^ F.^35^ Values of activation energy of total conductivities were calculated according to Arrhenius law using the equation:^36^1
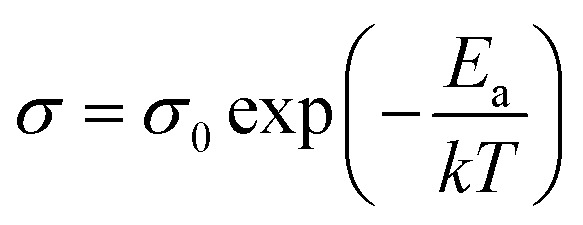
where *σ* is a total electrical conductivity, *T* – temperature, *σ*_0_ – pre-exponential factor, *E*_a_ – activation energy and *k* – Boltzmann constant.

### 2.4. Thermogravimetric analysis

Thermogravimetric analysis (TGA) was performed using Netzsch Jupiter® 449 F1 both in dry and wet atmospheres to analyse samples' oxidation and the water uptake. Before each measurement, a reference run for a baseline correction was carried out in both atmospheres separately. To analyse the oxidation, the sample mass change was recorded in dry synthetic air in the temperature range of 40–1000 °C. To measure the water uptake, a series of TG measurements with switches between dry and wet air was performed. To introduce water into the chamber, a gas mixer equipped with humidifying and drying stages was used. During the measurements, samples were heated up to 800 °C and held at this temperature for 3 h in dry synthetic air to remove adsorbed water and carbon dioxide from the surface. Next, samples were cooled to 300 °C in dry air. After two hours, the dry air (*p*_H_2_O_ ≈ 5.0 × 10^−5^ atm.) was switched into the humidified synthetic air (*p*_H_2_O_ ≈ 10^−2^ atm.). The difference between the masses recorded in the dry and wet atmospheres at 300 °C (Δ*m*_H_2_O_) allowed to estimate the molar protonic defect concentration 
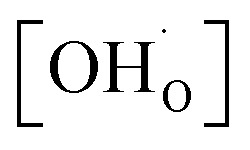
. As hydrogenation is a predominant process of forming protonic defects in strontium ferrite and titanate materials, the relation between Δ*m*_H_2_O_ and 
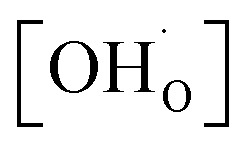
 is given by equation:^24^2
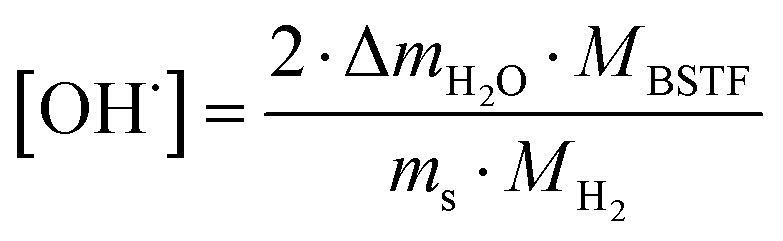
where *M*_BSTF_ and *M*_H_2__ are the molar masses of Sr_1−*x*_Ba_*x*_Ti_1−*y*_Fe_*y*_O_3−*δ*_ and molecular hydrogen respectively, whereas *m*_s_ is the mass of the sample in dry air before the switch to the wet gas.

## 3. Results

### 3.1. Crystal structure

**Fig. 1:**
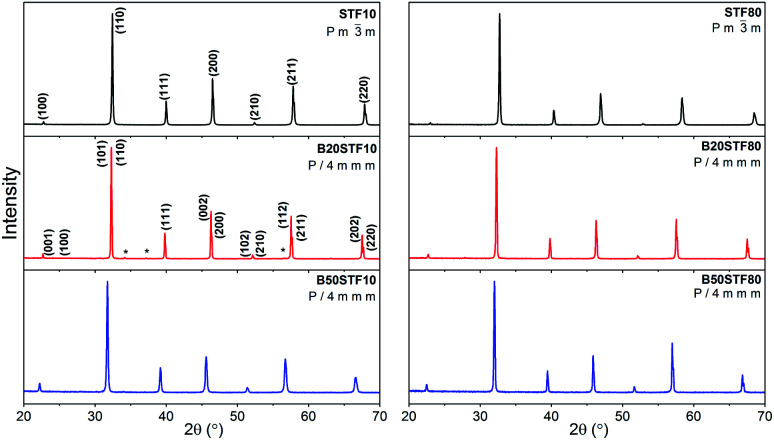
X-ray diffractograms of: STF10, B20STF10, B50STF10, STF80, B20STF80 and B50STF80 powders. Black stars mark the reflections of the Ba_0.5_Sr_0.5_Fe_12_O_19_ secondary phase.

Results of X-ray diffraction measurements are presented in Fig. 1. All synthesized samples except B20STF10 were obtained as single-phase perovskites. Barium substituted oxides (B*X*STF*Y*) crystallise in a tetragonal structure (*P*4/*mmm*, space group no. 123) while these without barium (STFX) crystallise in a cubic structure (*Pm*3̄*m*, space group no. 221). In B20STF10, a small amount of Ba_0.5_Sr_0.5_Fe_12_O_19_ (ICDD: 00-051-1879, *P*6_3_/*mmc*, space group no. 194) has been identified.

**Fig. 2:**
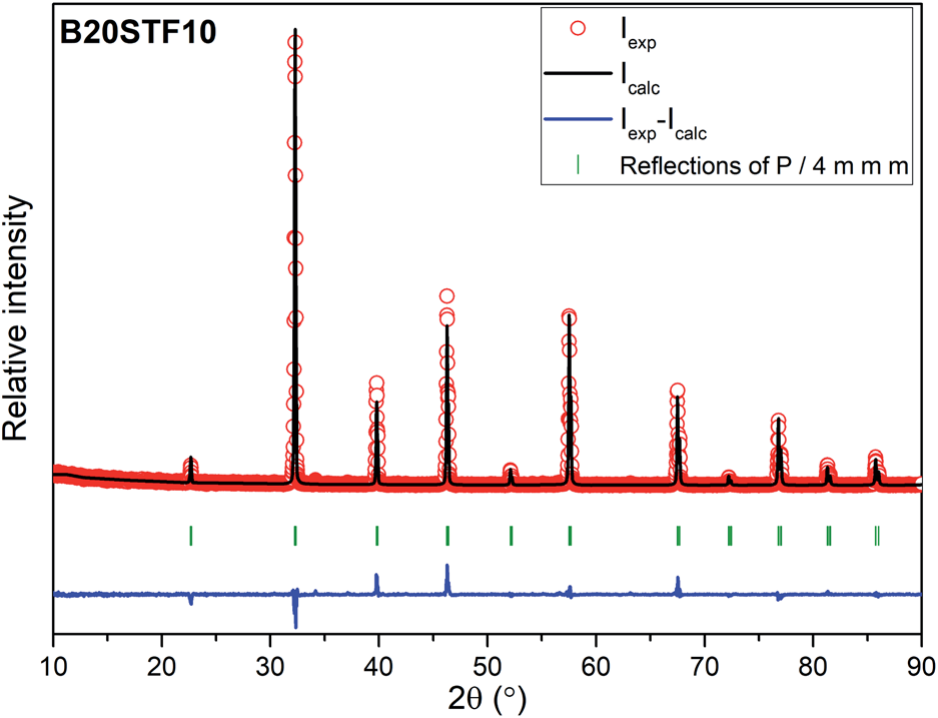
The exemplary profile of the Rietveld refinement obtained for B20STF10 sample.

The exemplary profile of the Rietveld refinement obtained for B20STF10 sample is presented in Fig. 2. The results of the Rietveld analysis for all samples are presented in Table 1.

**Table 1:** Selected structural parameters, theoretical densities (*ρ*) and relative densities (*ρ*_REL_) for Ba substituted Sr(Ti,Fe)O_3_ samples. Goldschmidt tolerance parameter (*t*) was calculated using 1.61 Å, 1.44 Å, 0.605 Å, 0.55 Å, 1.40 Å ionic radii of Ba^2+^, Sr^2+^, Ti^4+^, Fe^3+^and O^2−^, respectively^37^

Sample	*a* (Å)	*c* (Å)	*V* (Å^3^)	*ρ* (g cm^−3^)	*ρ*_REL_ (%)	*t*	*R*_wp_ (%)
STF10	3.913(1)	—	59.91(1)	5.000	91.2	1.0043	14.3
B20STF10	3.922(1)	3.920(1)	60.31(1)	5.348	88.1	1.0164	17.1
B50STF10	3.963(1)	3.956(1)	62.16(1)	5.588	87.8	1.0344	11.3
STF80	3.874(1)	—	58.13(1)	5.314	91.8	1.0241	15.2
B20STF80	3.9211(10)	3.9210(10)	60.29(1)	5.503	97.5	1.0363	14.3
B50STF80	3.946(1)	3.944(1)	61.400(1)	5.807	95.3	1.0547	17.5

Calculated unit cell parameters correspond well to previously reported both for pure and Ba-substituted Sr(Ti,Fe)O_3_ systems.^14–16,26,27,32^ As can be noticed, the unit cell parameters increase with increasing content of barium in the samples containing 10- and 80% of iron.

### 3.2. Microstructure

**Fig. 3:**
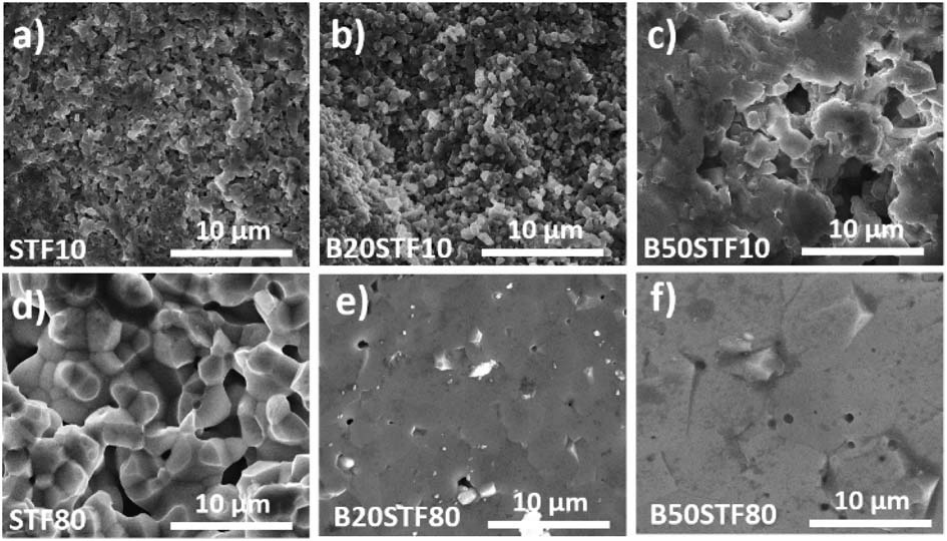
SEM micrographs of the cross sections of (a) STF10; (b) B20STF10; (c) B50STF10; (d) STF80; (e) B20STF80; (f) B50STF80 obtained with secondary electrons using ETD detector.

SEM micrographs obtained are shown in Fig. 3a–c and d–f, respectively for Ba-substituted SrTi_0.9_Fe_0.1_O_3−*δ*_ and SrTi_0.2_Fe_0.8_O_3−*δ*_. It can be seen that the microstructure of ceramics depends on the barium and iron content. The microstructure of STF10, B20STF10 and B50STF10 is typical of porous ceramics, however, the grains seen in the SEM image of B50STF10 are significantly larger in comparison with those of STF10 and B20STF10. Moreover, the porosity of B50STF10 appears to be lower. In the case of STF80, B20STF80 and B50STF80 series, the sample without barium (STF80) is porous, while the others are dense with only a few isolated pores seen in the images. The micrographs obtained with BSED revealed no phase contrast in the fractures of the examined materials (not shown).

**Fig. 4:**
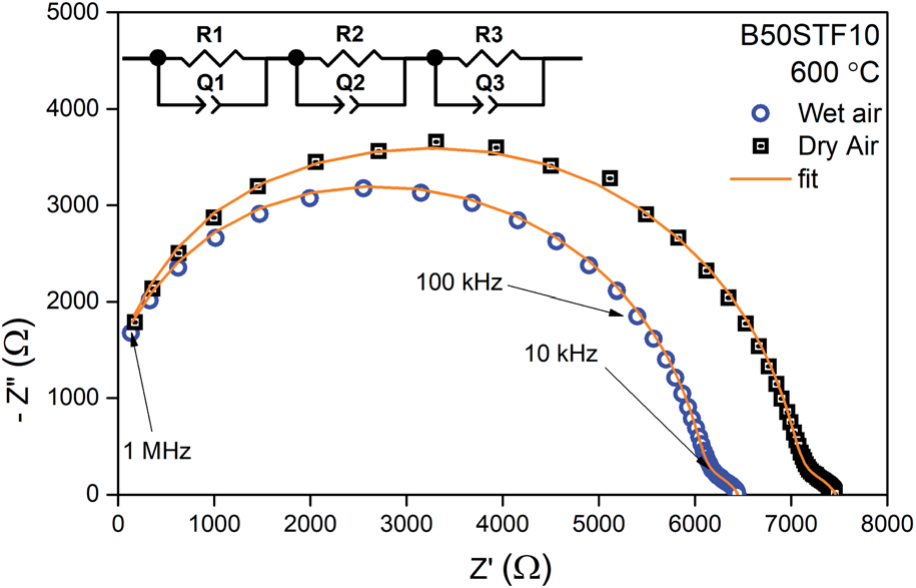
Exemplary EIS data together with the fit for B50STF10 at 600 °C in dry and wet air.

### 3.3. Electrical properties

**Fig. 5:**
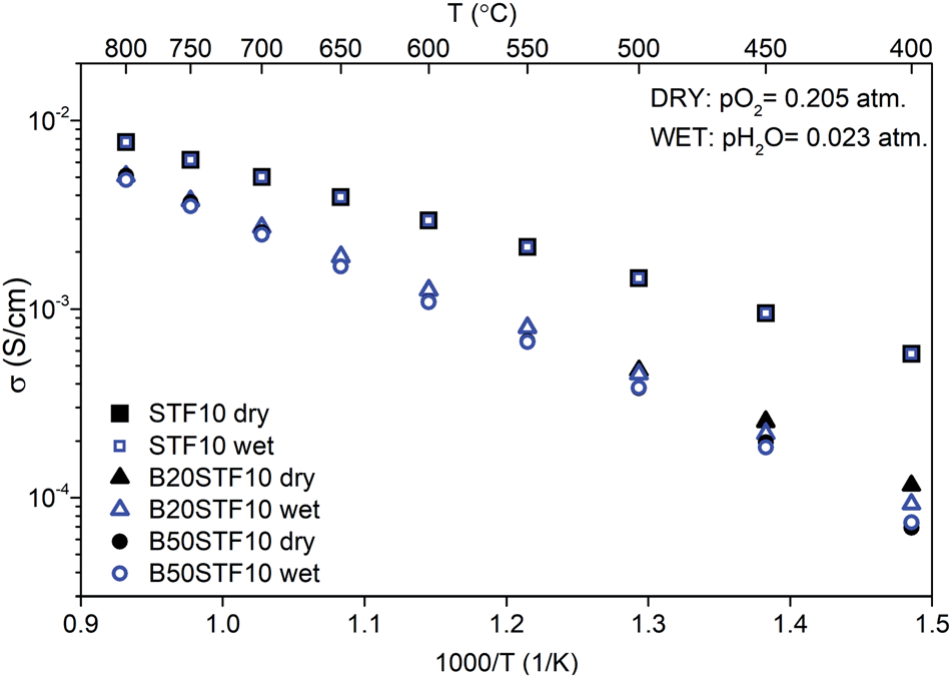
Temperature dependence of total conductivity of STF10 and BXSTF10 in dry and wet air.

Exemplary EIS data together with the fit for B50STF10 are presented in Fig. 4 whereas the temperature dependence of the total conductivity of the BXSTF10 samples is plotted in Fig. 5. It can be seen that the plot is linear, which means that it fulfils Arrhenius equation and, in the studied temperature range, conduction occurs through thermally activated processes. The highest conductivity was observed for STF10 whereas the lowest – for B50STF10.

The observed differences between conductivities in dry and wet air are small and they decrease with increasing temperature. The activation energy of total conductivity was calculated from the results in the high-temperature region (500–800 °C) using eqn (1). This temperature regime was chosen because below 500 °C a small nonlinearity of *σ*(1/*T*) function was observed. The results are collected in Table 2. In the sample with a higher content of barium, the higher activation energy of conductivity was observed. The presence of water vapour in the atmosphere hardly influenced the activation energy of conductivity. Only for B50STF10, a small difference between the activation energies in wet and dry air may be seen.

**Table 2:** Conductivity at 600 °C and activation energies of conductivities in dry- and wet air

Sample	*σ*_dry air_ (S cm^−1^)	*σ*_wet air_ (S cm^−1^)	*E*_a_ dry (eV)	*E*_a_ wet (eV)
STF10	2.9 × 10^−3^	3.0 × 10^−3^	0.41	0.40
B20STF10	1.3 × 10^−3^	1.3 × 10^−3^	0.56	0.57
B50STF10	1.1 × 10^−3^	1.1 × 10^−3^	0.62	0.60
STF80	14	14	—	—
B20STF80	0.8	0.9	0.32	0.30
B50STF80	1.6	1.0	0.12	0.23

**Fig. 6:**
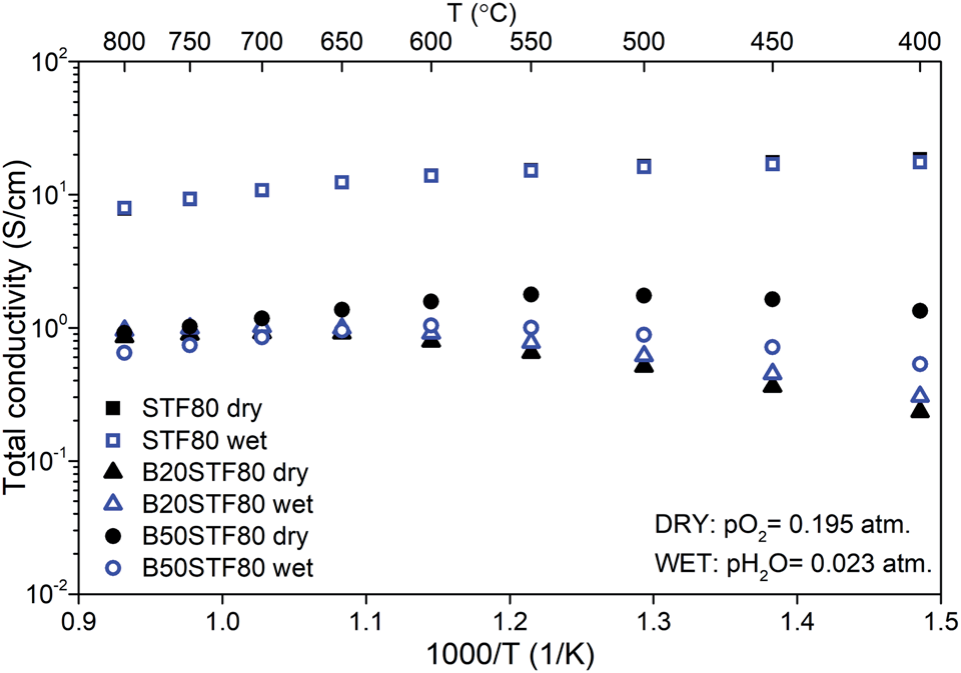
Temperature dependence of total conductivity of STF80 and BXSTF80 in dry and wet air.

The temperature dependencies of the total conductivity of BXSTF80 are presented in Fig. 6. For STF80, the total conductivity decreases with increasing temperature whereas for B20STF80 and B50STF80 it first increases, attains the maximum value and starts to decrease, therefore a metal–semiconductor transition is observed. The temperature range in which the temperature dependence of total conductivity changes between increasing and decreasing type depends on the barium content. For B20STF80 and B50STF80 it is around 600–650 °C and 550–600 °C, respectively. For STF80, in the studied temperature range, we did not observe the transition into a semiconductor state, but the transition temperature may be expected below 500 °C.^38^ All samples exhibit relatively high conductivity, the highest conductivity was observed for STF80 (14 S cm^−1^ at 600 °C). The presence of water vapour in the atmosphere influences the conductivity of samples containing barium, while for STF80 the difference was not observed. The difference between the total conductivities in dry and wet air increases with decreasing temperature. In B50STF80, the conductivity in dry air is higher than that in wet air, whereas the total conductivity of B20STF80 measured in wet-was higher than in dry air.

Activation energies of total conductivity were calculated from the data below the metal–semiconductor transition using eqn (1). The highest value of activation energy of total conductivity (0.42 eV) was found for B20STF80 in dry air.

### 3.4. Thermogravimetric analysis

Fig. 8a presents the relative mass changes recorded at the isothermal switch from dry to wet atmosphere at 300 °C. The process of mass increase in STF10 and B20STF10 occurs in a one-step, lasting about 10 minutes, and the relative mass changes observed at the switch are about 0.004% and 0.023%, respectively. On the other hand, the mass of B50STF10 first, similarly to STF10 and B20STF10, increases in a few-minute step of approximately 0.011%, then the kinetics of the mass changes slows down and the mass gradually increases with time. In the case of BXSTF80 samples, the mass changes are observed both before and after the gas switch. Moreover, the mass increase after the switch, similarly to B50STF10, occurs in two stages with different kinetics. The values of the mass increases observed in the first step are very low. The exemplary results for dry–wet–dry TG were presented in Fig. 8b. As one can see, after the backward from wet to dry atmosphere the mass decreases what indicate the release of protons from the structure. Moreover, in the 2nd dry regime a significant change of slope can be observed, what may be related to the influence of protons in the structure on the oxidation enthalpy.

**Fig. 7:**
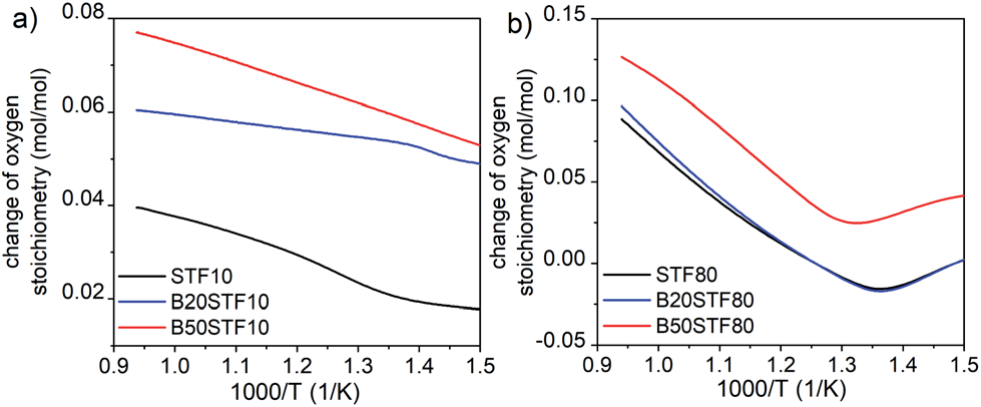
The change of oxygen stoichiometry recorded in the air during heating for (a) STF10 and Ba-substituted STF10 samples; (b) STF80 and Ba-substituted STF80 samples.

**Fig. 8:**
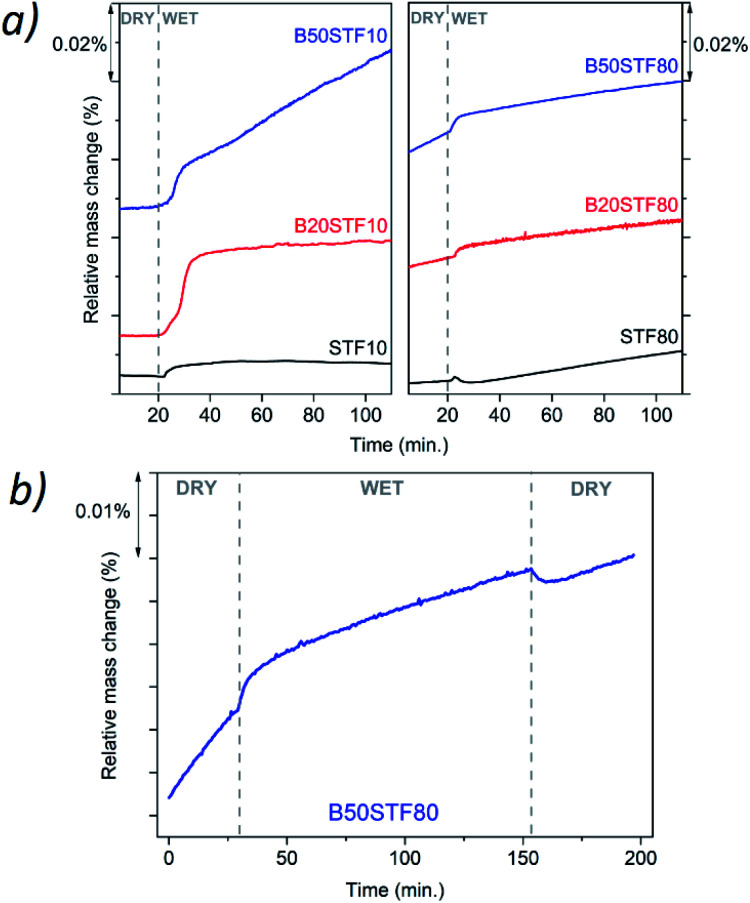
(a) Mass change recorded for all samples after isothermal switch at 300 °C between dry (*p*_H_2_O_ ≈ 3 × 10^−5^ atm.) and humidified air (*p*_H_2_O_ ≈ 2 × 10^−2^ atm.); (b) mass change recorded for B50STF80 sample after isothermal switch at 300 °C between dry and humidified air and between humidified and dry air.

## 4. Discussion

A solid solution of SrTiO_3_ and SrFeO_3_ is a mixed ionic–electronic conductor in which the contribution of electronic charge carriers is higher than that related to oxygen ions. The aim of introducing barium into this solid solution was to render it also a proton conductor. Therefore, the discussion will be focused on the influence of barium on the properties relevant to electrical properties. It includes structural and microstructural properties, water uptake, and oxidation processes as well as their relations with the concentration and mobility of particular charge carriers.

The structural changes are related to the higher ionic radius of barium (1.61 Å) compared to the ionic radius of strontium (1.44 Å).^37^ Since barium is isovalent with strontium, substitution with barium does not lead to the formation of other defects. On the other hand, barium can evaporate during the high temperature calcination, which may lead either to a barium deficiency or secondary phases formation. We believe that barium evaporation occurred in the case of the B20STF10 sample, in which a small amount of iron-rich Ba_0.5_Sr_0.5_Fe_12_O_19_ secondary phase formed. Nevertheless, increasing barium content in both series of materials led to the growth of unit cell volume. The difference between the B50STF10 and STF10 unit cell volume (2.25 Å^3^) is lower than that for B50STF80 and STF80 (3.27 Å^3^), which means that the bond strength in the BXSTF80 is lower than in BXSTF10. This is consistent with other literature data, where generally the Fe–O bond is reported to be weaker than Ti–O.^39^

Another structural change caused by the barium substitution is the tetragonal distortion of the unit cell. The tetragonal distortion caused by the introduction of the larger barium is higher in the BXSTF10 series of samples. This result is in contrast with the expectations since the Goldschmidt tolerance parameters for the BXSTF80 samples are higher than those for BXSTF10, so that it is the BXSTF80 samples that should exhibit tetragonal distortion of the unit cell. It should be noted that the unit cell parameters obtained for B50STF10 are in good agreement with the data calculated and shown by Guo *et al.*, where the influence of iron doping in Ba_0.5_Sr_0.5_TiO_3−*δ*_ was examined.^32^

The structural changes, that is, the growth of unit cell parameters, may result in changes in the electronic properties of the oxide. Unit cell parameters are related to the size of (Fe,Ti)O_6_ octahedra. Although the XRD results contain indirect information about the unit cell volume, but they provide some hints for the structural changes resulting from barium presence. In particular, in the BXSTF10 and BXSTF80 series, the estimated distance between the central atom and oxygen increased from 1.957 Å to 1.982 Å in BXSTF10 and from 1.937 Å to 1.973 Å in BXSTF80. This is in good agreement with literature data for similar compositions, for instance, XANES studies performed by Raimondi *et al.* shown that Fe–O distance increase from 1.95 Å in SrFeO_3_ to 1.97 in Ba_0.5_Sr_0.5_FeO_3_.^25^ Such a change in length corresponds to the weakening of the bond between B-cation and oxygen and may play a major role in the electronic structure of the material. The effect of this process was previously observed *i.a.* by Kuhn *et al.*^27^ They show that with an increase of a barium content, the decrease of bandgap energy and enthalpy of reduction occurs.^26,27^ The influence of elongating of Fe–O bonds on the conductivity of the material was examined by Hombo *et al.* in studies of SrFeO_3−*δ*_ and BaFeO_3−*δ*_. They show that the change of length of the Fe–O bond from 1.93 to 2.00 Å by substituting Sr by Ba leads to an overall decrease of the conductivity.^40^

Apart from the structural properties, also the microstructure was affected by the barium content. Higher barium content led to larger grains and lower porosity of the ceramics. Studies on BaTiO_3_–SrTiO_3_ solid solution formation showed that it proceeds with the preferential diffusion of Ba^2+^ and, around 1300 °C, with liquid phase formation.^41^ In our work, the sintering temperature did not exceed 1100 °C, so that the liquid phase did not form, however, taking into consideration the fast barium diffusion, the B50STF10 and B50STF80 compositions may be expected as more prone for easy densification. It should be also noted that the iron content increases the sinterability of these perovskites.^42^

To analyse whether the proton conduction is possible in the barium-substituted strontium titanate–ferrite solid solutions, proton defects formation should be discussed. In the air, in acceptor-doped SrTiO_3_, the concentration of oxygen vacancies is much lower than that of electronic holes. For example, as it was shown in^24^ at 400 °C in air, in SrTi_0.7_Fe_0.3_O_3−*δ*_, the molar oxygen vacancy equals 0.002, while the hole molar concentration is as high as 0.296. For all iron-substituted strontium titanates, proton incorporation was analysed and the most suitable mechanism was hydrogenation. It should be noted that the increased iron content led to a lower concentration of proton defects.^28^ Barium is isovalent with strontium, hence substitution with Ba does not introduce oxygen vacancies. Thus, also for BXSTF10 and BXSTF80 the hydrogenation, described by eqn (3) is the predominant reaction of proton defect formation.3
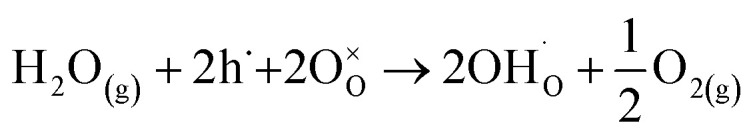


Molar proton concentrations calculated for BXSTF10 are presented in Table 3. The highest calculated value was 5.0 × 10^−2^ mol mol^−1^ for B20STF10. In contrast to expectations, for B50STF10 the molar proton concentration is lower than that for B20STF10. In this oxide, another process, seen in Fig. 8a as a gradual, almost linear with time mass increase, competes with the hydrogenation. We believe it is associated with oxidation which may be enhanced by the presence of water vapour. Similar mass change characteristics in humidified air, interpreted as the hydration/hydrogenation followed by oxidation, were observed in double perovskite cobaltites.^43^ It has to be underlined here that the concentration of proton defects was calculated only from the first few-minute step of the TG curve where the mass increases by approximately 0.011%. In the case of BXSTF80, due to the observed oxidation of the samples before the switch from dry to wet air which signifies the unknown oxygen stoichiometry, the molar proton concentration could not be determined. Nevertheless, it is significantly lower than that observed for the BXSTF10 samples. This is in agreement with the results obtained for SrTi_1−*x*_Fe_*x*_O_3_, where for the oxides with high iron content a very low concentration of proton defects was found and interpreted as caused by the high concentration of mobile electronic-type charge carriers.^24^ Even though the mass increase related to the hydrogenation of BXSTF80 is low, it can be seen that it is larger in the samples containing a larger amount of barium.

**Table 3:** Proton defects concentration for Ba doped STF10 samples at 300 °C

Sample	STF10	B20STF10	B50STF10
[OH˙] (mol mol^−1^)	8.5 × 10^−3^	5.0 × 10^−2^	1.9 × 10^−2^

Summing up, TG measurements showed a larger mass change in the samples containing barium. Such behaviour was previously observed for perovskite materials, where the increase of the water uptake was connected with an increase of the basicity of cations.^21,24,28,29^ Proton is bonded to oxygen belonging to the B–O bond, however, the role of the A-cation is important. For perovskites (ABO_3_), the influence of electronegativity of the A cation on the B–O bond was previously observed. Etorneau *et al.* point out, that the strength of the interaction between the A–O and B–O bonds, which is based on the average electronegativity of each sublattice and the formal valency of the A cation, can be related to the inductive effect of A on the B–O bonds.^44^ Since oxygen ion shares its electrons with the A- and B-cations, decreasing the electronegativity of A means that more electron density is available for the B–O bond. This may influence the water uptake.^24^ Zohourian *et al.*^21^ investigated the water uptake in perovskite materials based on Zn-doped (Ba,La)FeO_3_. They show that not only the A and B-site cations electronegativity plays a major role but also the average electronegativity of the A and B cations as well as defect interactions between protons and holes are essential. Sharing of electron pairs between atoms in the B–O bond corresponding to some delocalization of the hole from the transition metal-to the oxygen ion, decreases the electron density on the oxygen. This, consequently, leads to a decrease in water uptake. Raimondi *et al.* used X-ray absorption spectroscopy to study the (Ba,Sr,La)FeO_3_ group of compounds.^25^ The studies of Fe pre-K-edge peak have revealed that the addition of Ba to SrFeO_3_ effectively increases the electron density on the Fe–O bond, which decreases the hole concentration of Fe and is beneficial for proton uptake. In the same study, the extended X-ray absorption fine structure measurement of Fe K-edge showed that the local arrangement plays a significant role in protonation. Buckling of B–O–B bonds in ABO_3_ perovskite leads to a decrease in B–O orbital overlap, which reduces hole delocalization and, in turn, promotes proton defect formation. In our study, the Ba addition led to a slight tetragonal distortion of the perovskite structure, which was observed as contracting of the unit cell in one direction (*c*-axis). It is challenging to determine the subtle variation of B–O–B angles in the perovskite structure by X-ray diffraction. This is because the standard XRD technique is not sensitive enough for detailed studies of the oxygen atomic coordinates. However, one may postulate that this distortion could be an effect of the B–O–B buckling along the *c* axis. This is in good agreement with the fact that the BXSFT10 compositions, which surprisingly have the highest tetragonal distortion also exhibit the highest water uptake.

It is important to note here that the ionic conductivity in oxides is generally higher in materials with a higher symmetry crystal structure. The reason for this is that in a high-symmetry structure more equivalent positions are available for ionic transport. This is also characteristic of proton-conducting perovskites,^45^ however, for oxides being mixed protonic conductors with dominating electron–hole conductivity the influence of lattice symmetry may be more complex. It seems that proton–hole interaction is a limiting factor for proton conductivity in these materials and therefore every crystal distortion which reduces hole delocalization on the O-sites may be beneficial for proton defects formation. In that sense, in mixed conductors, lower symmetries can promote H^+^ conductivity, contrary to materials dominated by ionic charge carriers.

Finally, the influence of barium substitution on the total electrical conductivity will be discussed. The total conductivity in dry air is a sum of electronic- and oxygen-ion conductivities, whereas in wet air the contribution of proton conductivity should be considered. In all cases, in the air, the electronic p-type mechanism of conduction dominates the total conductivity.^14^ Mechanisms of electronic conduction that should be considered are band-type conduction and small polaron hopping between the localised Fe^4+/3+^ positions. STF perovskites containing either a low or high content of iron exhibit band-type conduction, while in the materials with intermediate iron content, both mechanisms were considered by different authors, *e.g.*^14,46,47^ Exact ranges of iron content corresponding to particular groups have not been determined. In the case of BXSTF10, we believe that the distance between Fe ions is too large for hopping conduction, therefore the band-type model will be considered. In the case of the conductivity of the BXSTF10 series in dry air, Sr-substitution with barium leads to a decrease of total conductivity, which is accompanied by an increase of activation energy of conductivity. As it was shown by Fleischer *et al.*,^48^ the temperature dependence of hole mobility in SrTi_0.98_Fe_0.02_O_3−*δ*_ is described by a power-law function with an exponent of −2.36. Similarly, Rothschild *et al.* in SrTi_0.75_Fe_0.25_O_3−*δ*_, SrTi_0.65_Fe_0.35_O_3−*δ*_ and SrTi_0.50_Fe_0.50_O_3−*δ*_ found the same temperature dependence of hole mobility with the exponent equal to −4.5, −3.4 and −4.4, respectively.^14^ The temperature dependence of conductivity in BXSTF80 is also affected by the barium content. Therefore, it may be assumed that in the B*X*STF*Y* oxides, the hole mobility decreases with temperature, which means that it is the temperature dependence of charge carrier concentration that determines the increase with temperature of the conductivity in BXSTF10. In SrTi_0.98_Fe_0.02_O_3−*δ*_ the temperature dependence of hole concentration was found to be described with typical of semiconductors exponential function with activation energy about 0.70 eV.^48^ The activation energy of conductivity in STF10 (0.47 eV) is consistent with that value since higher iron content leads to the broadening of the acceptor band which results in a decrease in activation energy.^49^ The barium presence causes an increase in activation energy as well as in the decrease in total conductivity.

In the case of the BXSTF80 samples, the content of iron is high, so that they exhibit metal-like conduction in either the whole or a part of the studied temperature range. However, the term metal-like conduction should be concerned only as an abbreviation for the conductivity decreasing with increasing temperature. In the case of oxides studied in this work, the reason for such a *σ*(*T*) relation may be a decrease with temperature of electron–holes concentration, while in metals it is decreasing electron mobility due to the phonon scattering process. The influence of barium presence on the total conductivity leads to a decrease in conductivity and the shifting of the temperature in which the temperature dependence of conductivity changes between increasing and decreasing, however, a systematic dependence on the barium content is not observed.

Summing up, in both BXSTF10 and BXSTF80 the observed changes in conductivity and its temperature dependence brought about by barium introduction cannot be directly related to Ba content since the barium presence influences both structural and electrochemical and electronic properties. Furthermore, Kim *et al.*^26^ reported that the relation between barium content and enthalpy of reduction, as well as bandgap energy for BXSTF35 materials with 0, 10 and 50 mol% barium substitution, is not monotonic. Nevertheless, since barium is isovalent with strontium, its presence does not directly affect the oxygen vacancy content. Barium presence was found to decrease the bandgap and reduction enthalpy.^26,27^ That leads to enhanced reducibility of barium-containing STF. Indeed, TG analysis at elevated temperatures in air revealed that mass loss is higher in the case of the samples with barium (see Fig. 7), however, the values of mass changes are not directly proportional to the barium content. This reduction is responsible for a decrease in hole concentration and total conductivity.

Finally, a possible contribution of proton conductivity will be discussed. Non-zero proton defects content was observed in B20STF10, B50STF10, B20STF80 and B50STF80. The influence of water vapour in the air on the total conductivity was observed in B20STF10, B50STF10 below 500 °C, B20STF80 and B50STF80 below 600 °C. The total conductivity of these samples apart from B20STF80 dropped in wet air. In the case of the materials with a high hole contribution to the total conductivity, a decrease of conductivity in wet air is caused by the competition between proton defects and holes. The protons' incorporation leads to a decrease in the concentration of electron holes. Since the mobility of holes is higher than that of protons, the total conductivity decreases. The opposite effect is observed for B20STF80, where a small increase in total conductivity is observed in a wet atmosphere. The total conductivity of this sample is lower, whereas the temperature in which the temperature dependence of conductivity changes between increasing and decreasing – higher than that of B50STF80. Even though the hole concentration in B20STF80 is lower than that in B50STF80, it is unlikely that it is the contribution of the proton conductivity which increases the total conductivity in the wet air. One of the possible ways to describe such behaviour is the creation of an additional charge carrier which is related to the oxidation of the material in wet atmospheres. According to the TG measurements, it was observed that in wet atmospheres the oxidation enthalpy is lower, which affects the hole concentration. As a consequence, electronic hole conductivity increased and affected the protonic conductivity which also increased. This hypothesis is in good agreement with the observed experimental results for the B20STF10 sample.

## 5. Conclusions

The samples of Ba_*x*_Sr_1−*x*_Ti_1−*y*_Fe_*y*_O_3−*δ*_ (where *x* = 0, 0.2, 0.5 and *y* = 0.1, 0.8) were synthesized by a solid-state route. The strontium substitution with barium caused structural and microstructural changes, that is, the unit cell volume and the grain size increased, whereas the ceramic porosity decreased with increasing barium content.

The proton defects formation was studied within the hydrogenation mechanism. It was shown that in the samples containing barium, proton defects are present in the wet air at the temperature of 300 °C. The highest proton defect concentration was 5.0 × 10^−2^ mol mol^−1^ for B20STF10. For BXSTF80, the molar proton concentrations were much lower than those observed for BXSTF10. The higher observed molar proton concentration for samples with barium is caused by its lower average electronegativity in comparison to Sr. The effect of barium on the total conductivity in dry air was discussed assuming that the electrical conduction is determined by the electron–hole band-type transport process. In both the BXSTF10 and BXSTF80 groups, barium introduction caused changes in conductivity and its temperature dependence, however, these changes were not directly related to the barium content. It was proposed that the main factor responsible for the barium-induced decrease of conductivity is the decrease in the electron–hole concentration related to the increased reducibility of the samples containing barium. The non-monotonic relation between the barium content and conductivity was explained by also non-monotonic relation between the barium content and reduction enthalpy. The contribution of proton defects to the total conductivity of B20STF10, B50STF10 B20STF80 and B50STF80 was indirectly observed through the influence of the wet air on the total conductivity.

## Author contributions

Conceptualization, T. M.; methodology, T. M., K. D., W. S.; validation, S. W., M. G.; formal analysis, M. G.; investigation, K. W.-P., D. J., T. M., P. W., A. M.-G., and K. D.; writing – original draft preparation, T. M., K. D.; writing – review and editing, T. M. and M. G. and all co-authors; supervision, M. G.; funding acquisition, M. G. All authors have read the manuscript and agreed to the published.

## Conflicts of interest

There are no conflicts to declare.

## Supplementary Material
